# The Jaw Adductor Resultant and Estimated Bite Force in Primates

**DOI:** 10.1155/2011/929848

**Published:** 2011-07-24

**Authors:** Jonathan M. G. Perry, Adam Hartstone-Rose, Rachel L. Logan

**Affiliations:** ^1^Department of Anatomy, Midwestern University, 555 31st Street, Downers Grove, IL 60515, USA; ^2^Departments of Biology and Anthropology, Penn State University, 3000 Ivyside Park, Altoona, PA 16601, USA

## Abstract

We reconstructed the jaw adductor resultant in 34 primate species using new data on muscle physiological cross-sectional area (PCSA) and data on skull landmarks. Based on predictions by Greaves, the resultant should (1) cross the jaw at 30% of its length, (2) lie directly posterior to the last molar, and (3) incline more anteriorly in primates that need not resist large anteriorly-directed forces. We found that the resultant lies significantly posterior to its predicted location, is significantly posterior to the last molar, and is significantly more anteriorly inclined in folivores than in frugivores. Perhaps primates emphasize avoiding temporomandibular joint distraction and/or wide gapes at the expense of bite force. Our exploration of trends in the data revealed that estimated bite force varies with body mass (but not diet) and is significantly greater in strepsirrhines than in anthropoids. This might be related to greater contribution from the balancing-side jaw adductors in anthropoids.

## 1. Introduction

Knowing the magnitude and orientation of the force produced by a muscle is critical to understanding muscle function. These variables can be used to better understand the properties of foods and how they relate to food-processing anatomy and behavior. They can also be used to provide more informed inferences about feeding behavior in fossils. The magnitude of muscle force can be estimated from the physiological cross-sectional area (PCSA) of the muscle. Orientation can be estimated from the positions of the muscle attachments. In a complex system where several muscles work together to perform a single action, knowing the vector of each muscle is critical to understanding function and adaptation in the system as a whole. The jaw adductors comprise such a system and the forces might be used as signals for dietary adaptation.

The jaw adductor muscles of primates work together to achieve food breakdown. This system consists of several muscular units that can be classified as the temporalis group (superficial, deep, and zygomatic temporalis), the masseter group (superficial masseter, deep masseter, and zygomatico-mandibularis), and the medial pterygoid [1–6]. Each muscle unit (e.g., superficial temporalis) has a different magnitude and orientation of pull. These can be summed into a single resultant for the entire jaw adductor system, and diet-related predictions can be made based on its orientation and magnitude. Such a resultant is only an approximation of the real resultant during mastication, for example, because of the possibility that different components of the chewing musculature experience peak activity at different times.

Greaves [7] used geometric models and measurements of mammalian skulls to generate hypotheses about the location and orientation of the jaw resultant. We endeavor to test these hypotheses here. In particular, Greaves generated the following hypotheses.

**Figure 1:**
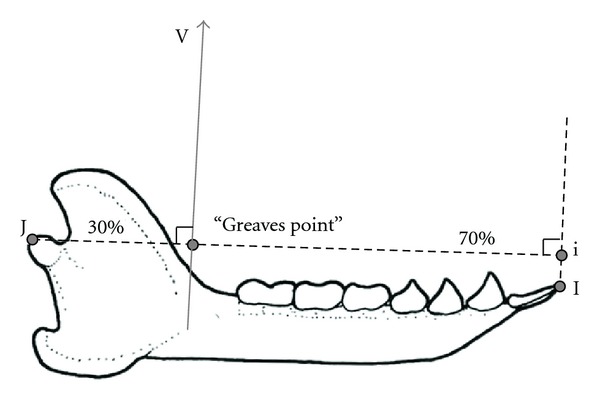
Diagrammatic representation of Greaves' model regarding the location of the resultant of the jaw adductors (V). In this drawing (of the mandible of *Varecia rubra*), the vector is positioned 30% of the way on the line J-i, where J is the posterior edge of the mandibular condyle and i is the projection of the anterior tip of the most anteriorly projecting incisors (I) onto the plane that is perpendicular to V and runs through J. Greaves hypothesized that this point would divide J-i into a posterior portion (30% of the length of J-i) and an anterior portion (70%). Also, V should lie just posterior to the last molar, which it clearly does not in this diagram. Here, the posterior edge of m3 lies at nearly 40%. Drawing by AHR.

1. The resultant crosses the mandible at approximately 30% of the distance from the temporomandibular joint (TMJ) to the anterior-most point on the dentition, measured perpendicular to the resultant (Figure 1). This hypothesis was based on integration calculations of the maximum average bite force across all bite points; Greaves found that average bite force reached a maximum at 30% [8].
2. The resultant crosses the mandible directly posterior to the posterior-most tooth [7, 9–12]. This hypothesis was based on the observation that maximum mechanical advantage for the resultant occurs at the greatest distance from the joint and that if the resultant were to pass anterior to any tooth, then biting on that tooth would load the TMJ in tension —a loading regime for which the joint is ill suited.
3. The resultant is inclined more anteriorly in mammals that do not need to resist great anteriorly directed forces [7]. These animals do not require a large temporalis (see [13]) and the masseter and medial pterygoid dominate, shifting the resultant anteriorly. This hypothesis can be applied to primates because some primate foods (e.g., some fruits, nuts, and seeds) likely resist incision more than others (e.g., most leaves) (see [14]).

Data on the location, orientation, and magnitude of the jaw adductor resultant can be used to estimate bite force magnitude when combined with measurements of the skull. One critical measurement is the distance between the TMJ and a given bite point (load arm). The resulting estimates of bite force can be evaluated experimentally using bite force transducers. In principle, predictions also can be made about how bite force varies with diet. Despite the wealth of data on primate food material properties published recently (e.g., [15–20]), at present, the material properties of primate foods are insufficiently quantified for us to generate specific predictions with regard to diet. This is partly because the published data focus on a small number of primates species, mostly outside the sample of available PCSA data. Rather, we perform a basic exploration of the data to detect correlations between estimated bite force and major diet category.

In this study, we evaluate the predictive power of Greaves biomechanical models for a subset of primate species. Because these models are based on an optimal configuration of the skull for producing bite force while minimizing tensile joint forces, the degree to which real primates conform to the models demonstrates the degree to which primate masticatory systems are optimally designed for biting and chewing. Our analysis of the resultant orientation tests the validity of the model for using resultant orientation to make predictions about diet. Lastly, we explore the data on estimated bite force for allometric, gross dietary, and gross taxonomic trends.

Previous studies have tested the validity of Greaves' biomechanical models (e.g., [21]) and many more have tested biomechanical models of the masticatory system in general (e.g., [13, 15, 22–29]). However, none of these were able to incorporate data on chewing muscle cross-sectional area because, until recently [30, 31], data on PCSA were not available for many primate taxa. In fact, Spencer stated that quantifying the chewing muscle resultant is hampered by the lack of data on comparative myology of primate chewing muscles. The availability of PCSA data allows us to increase the realism of muscle resultant estimates.

## 2. Materials and Methods

We used published data on jaw adductor PCSA for all taxa. These data come from two sources: one for the strepsirrhines and tarsiers [30, 32, 33] and one for the anthropoids [31]. Other data on primate chewing muscle PCSA have been collected by others using slightly different methods [34–36]. We have not used those data here because they pertain mostly to large catarrhines which are mainly absent from our current sample. 

For all data collection, our sample was divided into three subsets.

### 2.1. Subset 1

This subset includes all the strepsirrhines (21 species) and tarsiers (one species). For this subset (Table 1), we used maps of the origin and insertion areas for all of the individual jaw adductor muscles. These were made by one of us (J. M. G. Perry, see [32]) during the course of dissections. The attachment maps were used to reconstruct the resultant vector location in the following manner.

Photographs of the skull of the individual dissected or of a like-sized conspecific were taken in standard orientation. For each muscle group (temporalis, masseter, medial pterygoid), the origin and insertion areas were drawn onto the photographs using Image J software (Rasband WS, Research Services Branch, National Institute of Mental Health, Bethesda, Md, USA.). Finer muscular divisions (e.g., *superficial* masseter) were not used because borders between adjoining units could not be reliably reproduced [38]. The centroid of the origin attachment area was joined to the centroid of the insertion attachment area, and the angle between the resulting line and the occlusal plane was taken to be the orientation of the vector for this muscle group. The occlusal plane here is actually a visual estimate of the postcanine occlusal plane based on a line of best fit to the cusps visible in labial view. This muscle group vector was scaled to the PCSA (as a proxy for force) of the muscle group and multiplied by a constant value of muscle force per unit cross-sectional area, 3 kg per cm^2^ [39]. The same procedure was carried out for each muscle group. Finally, the horizontal and vertical components of all three muscle groups were summed to generate the resultant of the jaw adductor system.

**Figure 2:**
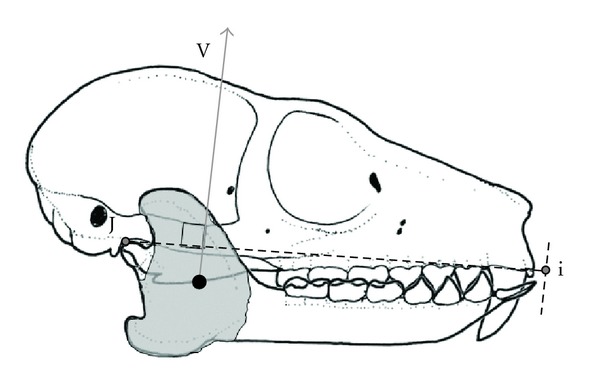
Method for anchoring the resultant vector. Symbols as for Figure 1. The shaded area on the mandibular ramus represents the common area of insertion for the jaw adductor muscles. The centroid of this area (black dot) was used to anchor the resultant vector. Note that, in this diagram, the vector is clearly well behind 30% of the distance from J to i. Drawing by AHR.

To position the resultant on the skull, we drew out the common insertion area for the jaw adductors. Apart from a small area surrounding the mandibular foramen and areas anteroventral to the TMJ, the entire mandibular ramus is covered by jaw adductor insertion on both its lateral and medial side. Making the unlikely assumption that jaw fibers are evenly distributed across this surface, we positioned the resultant at the centroid of this area (Figure 2). We drew a line extending superiorly from this point onto the photograph in ImageJ. This line constitutes the resultant and was drawn such that it intersects the occlusal plane at the calculated resultant angle (Figure 2). The distance from condylare (a point in the middle of the posterior-most edge of the mandibular condyle) to the anterior tip of the anterior-most incisor, taken perpendicular to the resultant, was measured; this is termed “Greaves Jaw Length” (GJL). The perpendicular distance from condylare to the resultant is here assumed to be equivalent to the moment arm of the jaw adductors (lever arm or moment arm of input force). The resultant, therefore, crosses GJL, dividing it into a portion posterior to the resultant and a portion anterior to the resultant. The percentage of GJL represented by the length of the posterior portion can be termed the Greaves Ratio and it is hypothesized to be 30% for all taxa (e.g., [12]).

Bite force can be estimated at any bite point given an estimate of the moment of the resultant and the length of the moment arm of the bite force (henceforth load arm). The moment of the jaw adductor muscle resultant itself is the product of the moment arm of the resultant (henceforth lever arm) and the magnitude of the resultant force. The load arm can be measured in several ways [5], for example, with the assumption of a vertical bite force [40–46]. Here we chose to assume that the TMJ is purely rotational, and therefore every bite point rotates in an arc for which the radius is equivalent to the absolute (shortest) distance from condylare to the bite point (see [24, 29]). The mandible rotates and translates simultaneously during mastication, and thus a much more complicated model is required to consider the effects of translation, partly because the instantaneous center of rotation must be found. Because the movement of the mandible near occlusion is likely mainly rotational, we have chosen to consider rotational forces only.

We chose two bite points: the posterior edge of the posterior-most lower molar (m3 or m2, depending on the taxon) and the anterior edge of the anterior-most incisor (usually i1). Some taxa might emphasize posterior bite force while others might emphasize anterior bite force. Estimating bite force at these two points allows us to evaluate anterior/posterior emphasis for our sample. All measurements and calculations were made in the lateral view, in a parasagittal plane.

All statistical analyses (described under Results) were performed in JMP 8.0.2 (SAS Institute Inc., 2009). For all regressions, we ran both least squares regressions and reduced major axis regression. Here, we report results for both only when they potentially lead to different conclusions.

### 2.2. Subset 2

This subset includes ten species of platyrrhines (Table 1). For this subset of our sample, we used microCT scans of skulls. These scans were rendered as three-dimensional surfaces in Avizo 6.0 (Visualization Sciences Group, 2009). Though more expensive to generate than photographs, CT scans are generally preferred because they likely reduce error in estimating the attachment sites for the medial pterygoid muscle. Jaw adductor muscle attachment maps do not exist for any of the anthropoids in our sample (Table 1). To determine the location and extent of each jaw muscle attachment area in anthropoids, we drew from our knowledge of strepsirrhines and tarsiers and from published anatomical depictions of these muscles in some anthropoids [34, 47–53]. 

**Figure 3:**
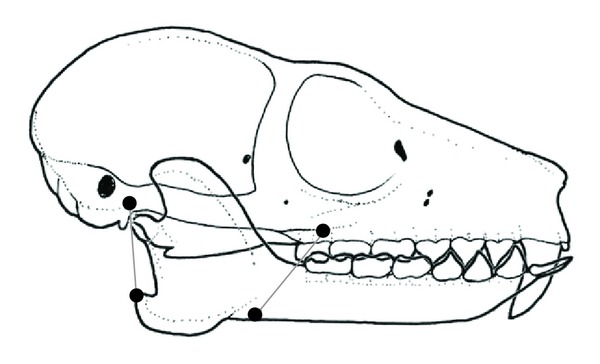
Method for determining the location and orientation of the masseter muscle for Subsets 2 and 3. The black dots on the cranium represent the anterior-most and posterior-most points of origin for the masseter muscle group. The black dots on the mandible represent the anterior-most and posterior-most points of insertion. The orientations of the two lines (relative to the occlusal plane) were summed to obtain the orientation of the resultant for the masseter. A similar process was applied to the temporalis and to the medial pterygoid. Drawing by AHR.

Rather than trying to delineate entire attachment areas, we used an anterior and posterior landmark for the origin and insertion of each muscle (Table 2). A line drawn from the anterior insertion point for the masseter to the anterior origin point for the masseter represents the vector for the anterior portion of the masseter (Figure 3). For each muscle group, the anterior and posterior vector were summed and then scaled by PCSA for that taxon. A value of one-half of muscle PCSA was assigned to the anterior part of each muscle and one-half for the posterior part. PCSA data were taken from Anapol et al. [31].

Calculations to determine the location, orientation, and magnitude of the resultant vector were the same as above for Subset 1. Calculations to estimate bite force also followed the procedure described above. All measurements and calculations were made in the lateral view, in a parasagittal plane.

### 2.3. Subset 3

This subset of analyses was added to extend our anthropoid sample beyond what was available to us in microCT scans. This sample includes four platyrrhine species and two catarrhine species (Table 1). For this subset, we performed all the procedures described for Subset 2. However, measurements were taken on lateral view photographs rather than microCT scans. The specimens used in this analysis were chosen from the mammalogy collections of the Field Museum of Natural History.

## 3. Results

### 3.1. Results Bearing on Greaves' Three Predictions

Earlier, we defined the ratio of the length of the jaw posterior to the resultant divided by the length of the jaw anterior to the jaw, expressed as a percentage, as the Greaves ratio (GR). Here, jaw length is measured perpendicular to the resultant. Greaves predicted that the resultant would lie at 30% of the jaw length [12], thus the predicted GR is 30% for all taxa in our study. In our sample of 35 primate species, the mean GR is 21.06% (Table 1). This value is significantly less than 30% (*P* < 0.0001). The mean for strepsirrhines is 20.46%, and the mean for anthropoids is 22.04%. The difference between the mean GR for the two taxa is not significant (Wilcoxon, *P* = 0.495). The value for the single species of tarsier is 19.21%. There are no significant differences in GR based on diet; see Table 1 for dietary categories. No species in the sample has a GR greater than 30%; the highest value is 27.75%, for *Alouatta fusca*.

Greaves also predicted that the posterior edge of the posterior-most molar would lie directly in front of the resultant [7, 9–12]. Thus, the posterior edge of the last molar should also lie at the GR (30%). We determined where the most posterior molar lies along Greaves jaw length (GJL) and expressed it as a percentage of GJL. The mean value is 42.74% for primates. Within primates, these values are 41.69% for strepsirrhines, 44.32% for anthropoids, and 41.55% for *Tarsius syrichta* (Table 1). The differences between taxa are not significant (*P* > 0.270). Given that on average the resultant is roughly 21% of jaw length from the condyle and the most posterior molar is roughly 43% of jaw length from the condyle, clearly the resultant is positioned well behind the last molar. In no species is the resultant closer than 13.07% of jaw length to the last molar.

To test Greaves' third prediction, that the resultant will be inclined more anteriorly in taxa that do not need to resist great anteriorly directed forces in feeding [7], we assigned each species to a dietary category based on published ecological data (Table 1). These categories are fruit, leaves, insects, gum, and fungi. We then performed an analysis of variance on resultant orientation using diet as the grouping variable. This analysis demonstrated that there are significant differences among dietary categories. Post hoc comparisons revealed that the significant differences in resultant orientation are between frugivores (mean = 93.75°) and folivores (mean = 85.33°). If fruits do provide more anterior resistance than leaves, then Greaves' third prediction is supported by our data on primates. Although gummivores might be expected to resist great anteriorly-directed forces when gouging trees to acquire gum, our results show no significant difference between gummivores and any other dietary group and the mean for gummivores is close to that for frugivores. However, given the small number of gummivores in our sample (two), this could be an effect of poor sampling. Nevertheless, in experimental conditions, callitrichid gummivores do not generate large forces while gouging for gum [54, 55].

The mean orientation of the resultant vector is 90.25° (89.10° in strepsirrhines, 91.93° in anthropoids, and 90.00° in *Tarsius syrichta*). The differences between taxa are not significant (*P* > 0.321).

### 3.2. Results for Estimated Bite Force

The magnitude of the muscle resultant ranged widely: from 10.20N in *Saimiri sciureus* to 555.34N in *Propithecus diadema *(Table 1). This wide range is unsurprising given the variation in body mass in our sample. Note that the value for *Hapalemur griseus* (62.78N) is within one standard deviation of the mean value (76.3N) reported from bite force transducer experiments at one-half of maximum gape [17].

To evaluate the possible relationship between posterior bite force (PBF) and diet in our sample, we performed an analysis of variance on PBF relative to body mass using diet as the grouping variable. We used the ratio of the square root of PBF to the cube root of body mass. For body mass, we used species mean values from Smith and Jungers [37]. The analysis of variance revealed no significant differences among diet groups (*P* = 0.1165). Individual comparison *t*-tests revealed that insectivores have significantly greater PBF (relative to body mass) than gummivores (*P* = 0.0298), and folivores have significantly greater PBF (relative to body mass) than gummivores (*P* = 0.0325). Only the first of these stands up to a sequential Bonferroni correction. The high mean for insectivores is unduly influenced by the high value for *Tarsius syrichta* and the difference between insectivores and gummivores disappears when this species is omitted from the analysis. At the subordinal level, there are no dietary differences in body-mass-scaled PBF among strepsirrhines (*P* = 0.1985) or among anthropoids (*P* = 0.6828). The ratio of PBF^1/2^ relative to body mass^1/3^ is not correlated with body mass^1/3^ either in raw space (*r* ^2^ < 0.0001, *P* = 0.9568) or in log space (*r* ^2^ = 0.0005, *P* = 0.9004). 

Reduced major axis regression of the bite moment arm at the posterior end of the dentition against the bite moment arm at the anterior end of the dentition yielded a very high correlation coefficient (*r* = 0.9771), and there are no clear outliers. Therefore, the data fail to support the hypothesis that some taxa emphasize strong posterior bites and others emphasize strong anterior bites. 

**Figure 4:**
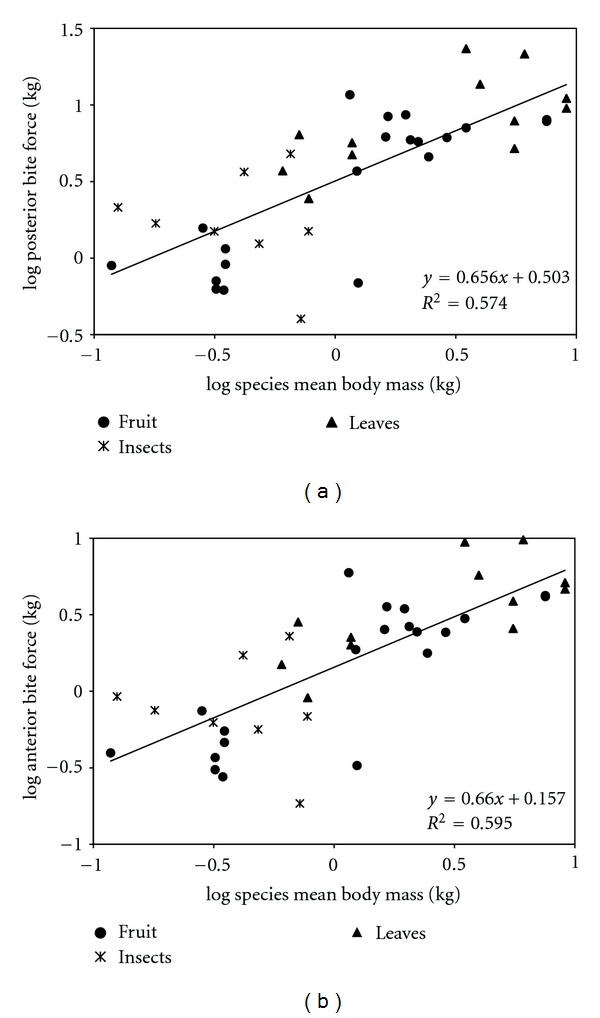
Least squares regressions of log_10_(estimated bite force) against log_10_(body mass) with the data grouped by broad dietary categories. See text for details. (a) Posterior bite force. (b) Anterior bite force.

We performed a least squares regression of base ten logarithms of PBF against base ten logarithms of body mass to evaluate the scaling relationship of bite force (Figure 4(a)). We did the same with log ABF (Figure 4(b)). In both cases, bite force scales isometrically with body mass (PBF slope = 0.656, intercept = 0.503, ABF slope = 0.660, intercept = 0.157). Reduced major axis regression suggests positive allometry; however, the 95% confidence interval of the slope includes 0.667 in each case (PBF slope = 0.857, intercept = 0.523, CI = 0.613–1.197, ABF slope = 0.845, intercept = 0.175, CI = 0.612–1.167).

**Figure 5:**
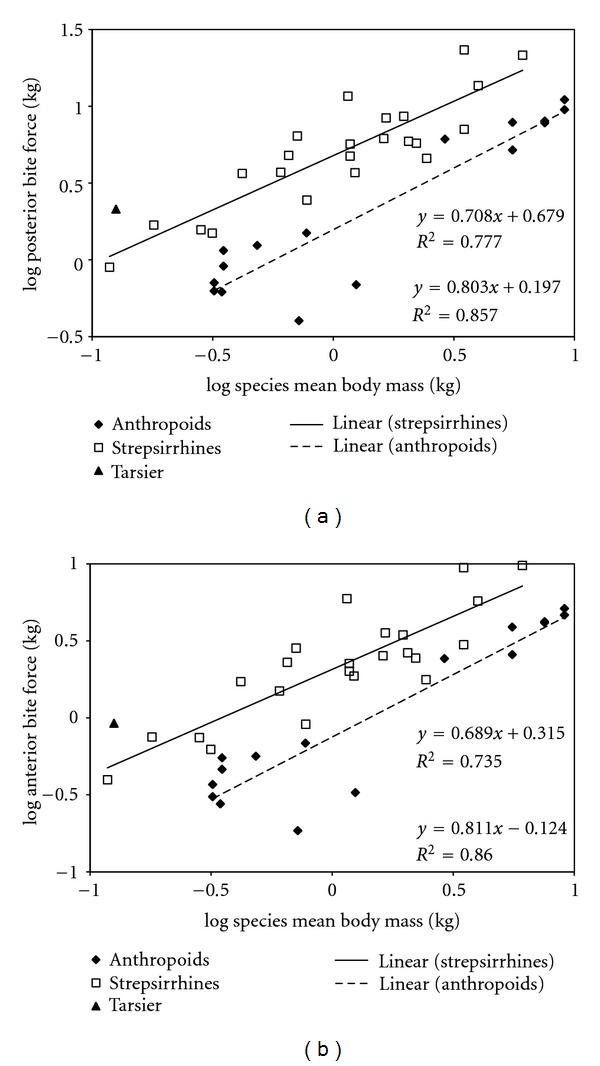
Least squares regressions of log_10_(estimated bite force) against log_10_(body mass) with the data grouped by major taxon. See text for details. (a) Posterior bite force. (b) Anterior bite force.

**Figure 6:**
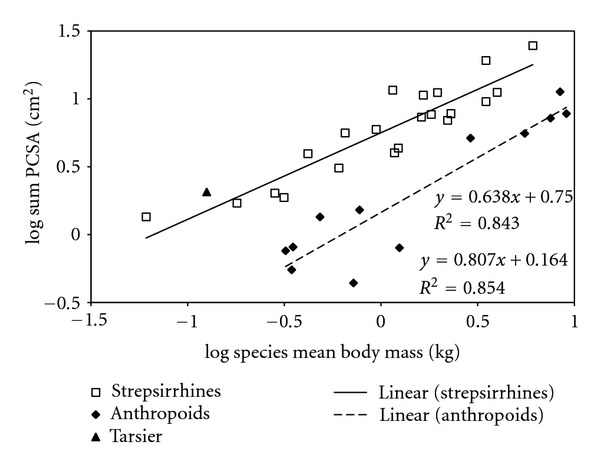
Least squares regressions of log_10_(sum PCSA) against log_10_(body mass) with the data grouped by major taxon. See text for details.

The total sample was divided by major taxon (strepsirrhines and anthropoids—*Tarsius syrichta* was excluded), and least squares regressions were computed for each taxon (Figure 5). This yielded the surprising result that the elevation of the line for strepsirrhines (PBF intercept = 0.679, ABF intercept = 0.315) is significantly higher than that for anthropoids (PBF = 0.197, ABF = −0.124). This difference is mainly an effect of a difference in PCSA between the two groups (Figure 6). The same effect was observed with reduced major axis regressions (PBF intercept: for strepsirrhines = 0.6986, for anthropoids = 0.1619; ABF intercept: for strepsirrhines = 0.3383, for anthropoids = −0.1662). We performed an analysis of covariance to determine if the slopes and intercepts for the LS lines of fit are significantly different. Although the slopes are not significantly different (*P* = 0.2548), the intercepts are (*P* < 0.0001).

## 4. Discussion

The primary goal of this study was to test three predictions regarding the jaw adductor resultant in primates. We also analyzed the variation in resultant orientation in the context of diet. Our secondary goal was to make a preliminary examination of the scaling of estimated bite force, using data on PCSA and skull shape. We also analyzed estimated bite force in the context of diet and major taxonomic group. In the following sections, we discuss the degree to which our observations did or did not conform to the three predictions. Then, we discuss variation in estimated bite force.

### 4.1. Predictions and Observations

The predicted position of the resultant was at 30% of Greaves jaw length from the condyle because, at this point, total bite force across all bite points should be optimized. Our results indicate that in, primates, the resultant is significantly posterior to the 30% point. Greaves' second prediction, that the resultant vector is directly behind the last molar, is also not supported by our data. 

Spencer [21] performed a test of several of Greaves' predictions regarding the position of the chewing muscle resultant. He used landmarks from a broad sample of anthropoid skulls to estimate the location of the resultant and assumed that the resultant is inclined at 80° from the occlusal plane (anterior to vertical). Spencer also found that his estimated resultant lays significantly posterior to the last molar, just as we found. He suggested two possible nonmutually exclusive explanations. First, selection against loading the TMJ in tension has been so strong in primates that the position of the resultant vector relative to last molar ensures a considerable factor of safety. Second, selection for wide gapes has been so strong in primates that the masticatory system favors excursion at the expense of bite force.

If true, the first explanation permits considerable asymmetry in muscle activity in favor of the anterior parts of the chewing musculature, without the resultant passing anterior to the posterior edge of the last molar. In this study, we have ignored electromyographic data on the jaw adductors despite the wealth of published data for some taxa. This is because considering EMG quantitatively would have required extensive extrapolation across taxonomic lines given the small number of primate species for which EMG data are available. Nevertheless, it is clear from the EMG literature that jaw muscle activity is extremely variable between taxa, and between individuals, trials, and chews in a sequence [56]. In this context, it is conceivable that primates could, in some situations, modulate muscle activity such that the resultant vector is shifted quite far anteriorly.

The explanation regarding gape seems much more compelling, partly because it is supported by the growing body of research that emphasizes the importance of gape adaptation in shaping the evolution of the primate skull [22, 54, 55, 57–60]. The lack of differences in Greaves Point with respect to diet suggests that all primates, regardless of diet, trade optimal bite force for increased gape. In addition to its noningestive functions [61], wide gape allows large foods to be introduced into the oral cavity at its anterior end. However, it can also be related to accommodating large objects at the back of the mouth. For example, if a large seed or nut is so hard that it can only be broken down using the heightened leverage at the molars, then a very wide gape is required at the *front* of the mouth to accommodate the unreduced food item at the *back* of the mouth.

Although our data do not support the first two predictions of Greaves, they do not invalidate the models. The data merely serve to demonstrate how the ecology of primates has shaped the evolution of their masticatory apparatus such that they deviate from the expectations of optimization.

The third prediction was upheld by our data, so long as folivores can be considered animals whose foods do not provide as much anterior resistance as the foods of frugivores. Given the extensive incisal preparation required for many fruits, this is a distinct possibility. Smith and Savage [13] suggested that the main advantage of the temporalis over the masseter and medial pterygoid is that it is ideally situated to resist anterior forces (e.g., the struggling movements of prey) and this provides one explanation of the large temporalis in carnivorous mammals. 

Greaves provided a rationale for why the masseter and medial pterygoid should be favored in the absence of anteriorly-directed resistance [7]. The masseter and medial pterygoid are inclined anteriorly while the temporalis is inclined posteriorly, such that when the former are larger relative to the latter, the resultant pitches anteriorly and when the latter is larger relative to the former, the resultant pitches posteriorly. If the lever arm of the resultant is held constant and the last molar must lie directly anterior to the resultant, then a posteriorly inclined resultant requires there to be more bone between the TMJ and the teeth than would an anteriorly inclined resultant. Thus, Greaves argued (from the standpoint of conservation of energy and mass) that increasing the sizes of the masseter and medial pterygoid at the expense of the temporalis is adaptively favorable.

The orientation of the resultant is significantly more anterior in folivores than in frugivores. Folivorous primates apparently emphasize the masseter and medial pterygoid over the temporalis. This finding is supported by the PCSA data [33]. Whether the masseter and medial pterygoid are emphasized in folivores because their foods provide less anterior resistance or, for example, because these muscles are ideally oriented to provide a forceful transverse chew is unknown. The heightened activity of the balancing-side deep masseter late in the chewing stroke provides correlative support for the latter [62–64], but we lack sufficient comparative EMG data on folivores and frugivores to test this hypothesis.

The mean resultant vector orientation in primates is 90.25°. Furthermore, there is only moderate variation around the mean (range = 76.77° to 102.53°). This suggests that primates are generalized in the configuration of the masticatory system relative to ungulates, rodents, and lagomorphs (dominated by the masseter and medial pterygoid) and relative to carnivorans (dominated by the temporalis) [5, 51]. This is unsurprising given the variability of primate diets. 

Spencer [21] suggested that the resultant in primates is probably rarely perpendicular to the occlusal plane. Our results suggest that a vertical vector is probably a reasonable approximation; however, the presence of variation in resultant orientation (including variation related to diet) should not be ignored. Spencer used an estimated resultant orientation of 80° for his analysis, based on data from bony landmarks. Our results suggest that this value, too, is a reasonable approximation.

### 4.2. Variation in Bite Force

There is no clear way in which estimated bite force varies in relation to diet. Primate diets are complex and varied. It may be that broad dietary categories like frugivory include foods with many different properties. Also, perhaps one particular insect is more like one particular fruit in its material properties than it is like some other insect. Given these complications, it might be difficult to detect a signal for dietary variation in bite force when using broad categories like frugivory and insectivory. Although there is an increasing dataset for material properties of primate foods [19, 65, 66], there is much to be done before broad species comparisons can be made between anatomical traits and food material properties [15].

The distribution of the data for posterior bite force almost exactly matches that for anterior bite force. Perhaps primates have not undergone changes in the proportions of the skull to emphasize posterior bite force over anterior bite force (or vice versa). It may be that there are subtle differences not detected in our gross comparison. These might appear if we compared pairs of closely related taxa that differ in diet (e.g., [67]). Admittedly, we did not evaluate the influence of phylogeny on our data. However, given the extremely high correlation between posterior bite force and anterior bite force, we doubt that the inclusion of phylogenetic corrections would greatly alter the result.

The great difference between strepsirrhines, and anthropoids in estimated bite force is particularly interesting. This is mostly an effect of a difference in PCSA; it suggests that PCSA of the jaw adductors of strepsirrhines is much greater compared to body mass than that of anthropoids. 

Could this difference be blamed on observer bias? The PCSA data for strepsirrhines were collected by one of us [32], while the anthropoid data were culled from a study by Anapol and colleagues [31]. Nevertheless, the study by Anapol et al. does include data from some strepsirrhines, and those strepsirrhines conform to the overall strepsirrhine pattern in having large jaw adductor muscles. Therefore, if present, observer bias is likely minimal and does not account for the large difference observed here.

One possible biological explanation for the large jaw adductors of strepsirrhines compared to anthropoids stems from the work of Hylander and colleagues [63, 68, 69]. They demonstrated that the balancing-side jaw adductors in strepsirrhines do not contribute as much to bite force production as they do in anthropoids. It was argued that this is because the unfused mandibular symphysis of strepsirrhines is less able to withstand particular chewing loads when both working-side and balancing-side muscles are highly active. Strepsirrhines provide an interesting test case for this hypothesis because the degree of symphyseal fusion varies among them [62, 63].

Thus, imagine a strepsirrhine (with an unfused symphysis) and an anthropoid (with a fused symphysis) that have the same body mass and are eating the same food that requires an input of 6N of chewing muscle force before it will fail. If the strepsirrhine receives one-fifth of that force from the balancing-side muscles and the anthropoid receives half from the balancing side (based on EMG and bone strain data), then the strepsirrhine provides 1N from the balancing side and 5N from the working side whereas the anthropoid provides 3N from the balancing side and 3N from the working side. This means that the anthropoid can break down the same food as the strepsirrhine, but with chewing muscles that are 3/5 the cross-sectional area.

### 4.3. Limitations

A few caveats are worth mentioning. First, it may be that our values for the Greaves ratio are low because our measurements were taken in a parasagittal plane, while primate jaws in occlusion do not lie in a parasagittal plane. However, the jaws of long-jawed primates are closer to being parasagittal than those of short-jawed primates. If the difference between our observed Greaves ratios and the predicted ratio of 30% was due solely to measurements out of plane, then we would expect there to be a relationship between jaw length and Greaves ratio: there is no such relationship.

Second, the PCSA data provided by Anapol et al. [31] are specified to genus level only. Therefore, there may be incorrect matching of our osteological data to their muscle data at the species level. Given the rarity of published PCSA data, there is little we can do about this short of carrying out many dissections of anthropoid chewing muscles.

Last, our sample size for each species is very small. There are PCSA data on only a few individuals per species. We have also confined our osteological sample to a single specimen per species. If the specimens chosen for either dissection or cranial analysis do not represent the norm for their species, then the results as a whole might be nonrepresentative. We endeavored to choose well-preserved specimens that are dentally adult, normal sized, and nonsenescent. Intraspecific variation is particularly problematic when there are dimorphs within the population (e.g., sexual dimorphs). Few of our taxa exhibit strong sexual dimorphism, but some exhibit very strong sexual dimorphism (e.g., *Alouatta fusca*).

## 5. Conclusions

Our data on primates suggest that the resultant vector of the jaw adductors passes significantly posterior to the 30% point, the location where average bite force across the teeth is maximized. Furthermore, the most posterior molar is located well anterior to that 30% point. These observations suggest that bite force is reduced in primates relative to the previously hypothesized optimum, perhaps as an extra precaution against TMJ tension and/or to allow for increased gape.

The resultant vector is inclined more anteriorly in folivores than in frugivores. This is due to large masseter and medial pterygoid muscles and/or small temporalis. Possible explanations for this difference in muscular emphasis include a lack of anterior resistance in the foods of folivores and the more effective lines of action of the masseter and medial pterygoid for producing transverse chewing forces.

Estimated bite force does not seem to vary with diet in primates, nor are there obvious cases of species that are specialized for either posterior or anterior bites. However, strepsirrhines and anthropoids differ markedly in estimated bite force relative to body mass. This seems to be due to significantly greater jaw adductor PCSA in strepsirrhines. Likely this difference is due to varying input from the balancing-side muscles depending on the presence of symphyseal fusion. Because strepsirrhines do not receive much force from the balancing-side muscles, the working-side muscles need to be capable of producing considerable force.

## Figures and Tables

**Table 1:** Primary data in this study.

Species	Diet	Body mass (kg)^a^	Greaves ratio (%)	Location of last molar (%)	Resultant orientation (degrees)	Muscle resultant force (N)	Posterior bite force (N)
SUBSET 1							
*Avahi laniger*	Leaves	1.18	24.07	39.45	77	92.41	55.62
*Cheirogaleus medius*	Fruit	0.28	21.55	46.77	86	34.24	15.40
*Eulemur collaris*	Fruit	2.05	19.73	41.25	95	143.32	58.08
*Eulemur coronatus*	Fruit	1.66	19.31	41.68	93	178.93	82.50
*Eulemur macaco flavifrons*	Fruit	2.44	14.23	36.33	99	130.87	44.93
*Eulemur mongoz*	Fruit	1.62	18.55	40.18	90	136.06	60.63
*Eulemur rubriventer*	Fruit	1.96	19.93	37.27	89	176.58	84.56
*Galago moholi*	Insects	0.18	21.71	45.25	83	34.53	16.58
*Hapalemur griseus*	Leaves	0.71	20.70	40.39	87	125.27	62.78
*Lemur catta*	Fruit	2.21	19.10	41.37	92	126.84	56.51
*Lepilemur leucopus*	Leaves	0.61	22.34	43.97	85	64.06	36.49
*Microcebus murinus*	Fruit	0.12	22.25	40.92	94	17.95	8.83
*Mirza coquereli*	Insects	0.32	18.04	41.45	90	34.43	14.72
*Nycticebus coucang*	Insects	0.65	25.70	46.09	99	118.01	46.99
*Nycticebus pygmaeus*	Insects	0.42	24.05	48.71	92	70.34	35.81
*Otolemur crassicaudatus*	Fruit	1.15	21.17	41.58	90	352.18	114.19
*Perodicticus potto*	Fruit	1.23	23.38	49.90	88	79.56	36.30
*Propithecus coquereli*	Leaves	3.99	19.42	40.08	88	283.21	134.00
*Propithecus diadema*	Leaves	6.10	18.82	34.71	86	555.34	211.21
*Propithecus tattersalli*	Leaves	3.49	21.15	43.10	85	440.67	228.87
*Varecia rubra*	Fruit	3.49	19.43	38.84	95	154.90	69.55
*Tarsius syrichta*	Insects	0.13	19.21	41.55	90	49.15	20.99
SUBSET 2							
*Alouatta fusca*	Leaves	5.54	19.52	34.93	91	140.38	51.01
*Aotus trivirgatus*	Fruit	0.77	19.80	41.08	93	35.02	14.72
*Ateles geoffroyi *	Fruit	7.54	24.52	44.49	90	176.29	78.68
*Callicebus torquatus*	Fruit	1.25	18.35	40.08	103	19.91	6.77
*Callimico goeldii*	Fungi	0.48	20.06	41.07	94	28.55	12.16
*Mico argentatus*	Gum	0.35	21.99	42.55	92	12.75	6.08
*Cebus nigrivittatus *	Fruit	2.91	22.02	35.09	99	113.89	60.04
*Saguinus fuscicollis *	Insects	0.35	26.37	47.27	87	20.60	11.28
*Saimiri sciureus*	Fruit	0.72	19.16	60.54	100	10.20	3.92
*Callithrix jacchus*	Gum	0.32	18.75	44.99	93	17.07	6.18
SUBSET 3							
*Alouatta fusca*	Leaves	5.54	27.75	44.26	71	141.36	77.20
*Ateles geoffroyi*	Fruit	7.54	23.75	48.00	98	176.48	76.62
*Callithrix jacchus*	Gum	0.32	23.10	50.00	93	16.28	6.97
*Colobus polykomos*	Leaves	9.10	22.73	44.32	86	213.17	93.39
*Colobus rufomitratus*	Leaves	9.10	19.81	39.63	93	276.74	108.40
*Saguinus fuscicollis*	Insects	0.35	24.94	50.82	87	18.44	8.93

^a^Body mass values are means from Smith and Jungers [37].

**Table 2:** Landmarks for each anterior and posterior resultant vector.

Muscle group attachment	Anterior landmark	Posterior landmark
Temporalis origin	Point where superior temporal line meets post-orbital bar	Point where superior temporal line meets nuchal crest
Temporalis insertion	Anterior corner of masseteric fossa (also anterior end of cristid obliqua of mandible)	Posterior-most point on the hook formed by the tip of the coronoid process
Masseter origin	Anterior zygomatic point	Posterior zygomatic point
Masseter insertion	Point on ventral border of mandibular corpus directly inferior to the anterior corner of the masseteric fossa	Posterior end of angular process of mandible
Medial pterygoid origin	Point where medial pterygoid plate meets lateral pterygoid plate	Posterior end of the inferior border of the lateral pterygoid plate
Medial pterygoid insertion	Anterior-most point within the medial pterygoid fossa	Posterior-most point within the medial pterygoid fossa
